# Two lucky survivors of thoracic impalement in childhood: case report and literature review

**DOI:** 10.1186/s12893-020-00790-z

**Published:** 2020-06-15

**Authors:** Samuel Negash, Tihitena Negussie Mammo

**Affiliations:** Division of pediatric surgery, Department of surgery, Addis Ababa University, Addis Ababa, Ethiopia

**Keywords:** Impalement, Penetrating injury, Chest injury, Thoracic injury, Case report

## Abstract

**Background:**

Penetrating thoracic injuries are uncommon in childhood. Massive penetrating injury due to impalement is even more rare and has scarcely been reported. It has a dramatic clinical presentation and is often fatal, depending on the organs injured.

**Case presentation:**

Two boys presented with an unusual mechanism of injury. They fell from a height to be impaled by a large stick. Appropriate emergency medical service was not available and surgery was delayed by more than 24 h after the accident. Both children were labelled “lucky” as they survived the injury without any significant sequelae.

**Conclusion:**

We discuss two new cases of pediatric thoracic impalement and perform the first literature review on the subject. Emphasis should be given to the initial care which comprises avoiding premature removal, rapid transport, resuscitation, anti-tetanus and antibiotics. All reported cases had a favorable outcome, even those managed within the constraints of low-income countries.

## Background

Penetrating thoracic injury is uncommon in children [1, 2]. It usually occurs in older boys while undertaking dangerous activities and can be more devastating than in adults [1–3]. Thoracic impalement is the severest form of these penetrating injuries where the object remains in the human body [4, 5].

Impalement injury is very rare in civilian practice [6, 7]. It is usually caused by large blunt objects made of wood (logs, branches) or metal (posts, poles and pipes). These accidents mostly occur in the setting of occupational or traffic accidents which are more common in adults [8–11]. Impalement in children is a rare and unique finding that merits discussion [2].

There are several concerns in the management of these injuries during prehospital, transportation, as well as in-hospital setting [5]. The different physiology in children adds to this challenge [1]. Furthermore, resource constraints in developing nations make it difficult to provide organized prehospital service and modern trauma care [12]. Nonetheless, the most vital principle has to be followed. Premature removal is fatal and should be avoided at all costs [4, 5, 8, 13, 14].

Here we report the case of two boys that came from the rural part of Ethiopia with a large stick impaled in the chest. We also discuss available literature on the subject. Considering the degree of impalement with the outcome, the children can be deemed extremely lucky.

## Case presentation

### Case 1

A 7 year old boy came to the pediatric emergency department 16 h after a penetrating thoracic injury. He fell down from a tree and got injured by a stick which was implanted in the ground. The family tried to remove it but were not succesful. He was then taken to a local health center which referred him to our hospital after chest tube inserted on the right side. He was given analgesics and started on antibiotics.

**Fig. 1:**
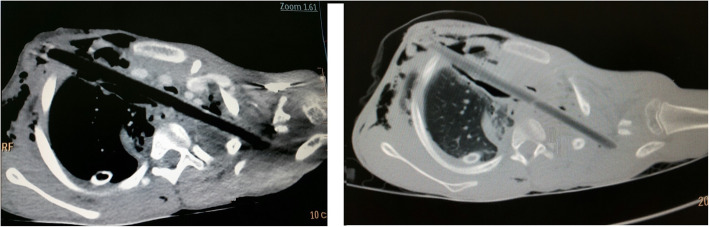
CT scan demonstrating the trajectory of the penetrating foreign body in the axial plane through the anterio-superior mediastinum, directed superiorly to the left side all the way to the medial edge of contralateral shoulder joint

Upon arrival to our center, examination revealed a huge stick impaled in his right thorax with a diameter of about 1.5 cm and about 10 cm length visible outside. It had entered the chest cavity directed diagnoally at the 2nd intercostal space and there was clavicular fracture on contalateral side but there was no exit. His vital signs were stable and he had good air entry bilaterally. He had no pain or difficulty during swallowing. He had no signs of other associated injuries. CT scan of the thorax showed the trajectory to traverse the superior mediastinum and penetrate the apex of the contralateral lung. There was no injury to great vessels or airways. (Fig. 1) We considered inserting a second chest tube on the left side but it was deferred as there was no hemopneumothorax.

**Fig. 2:**
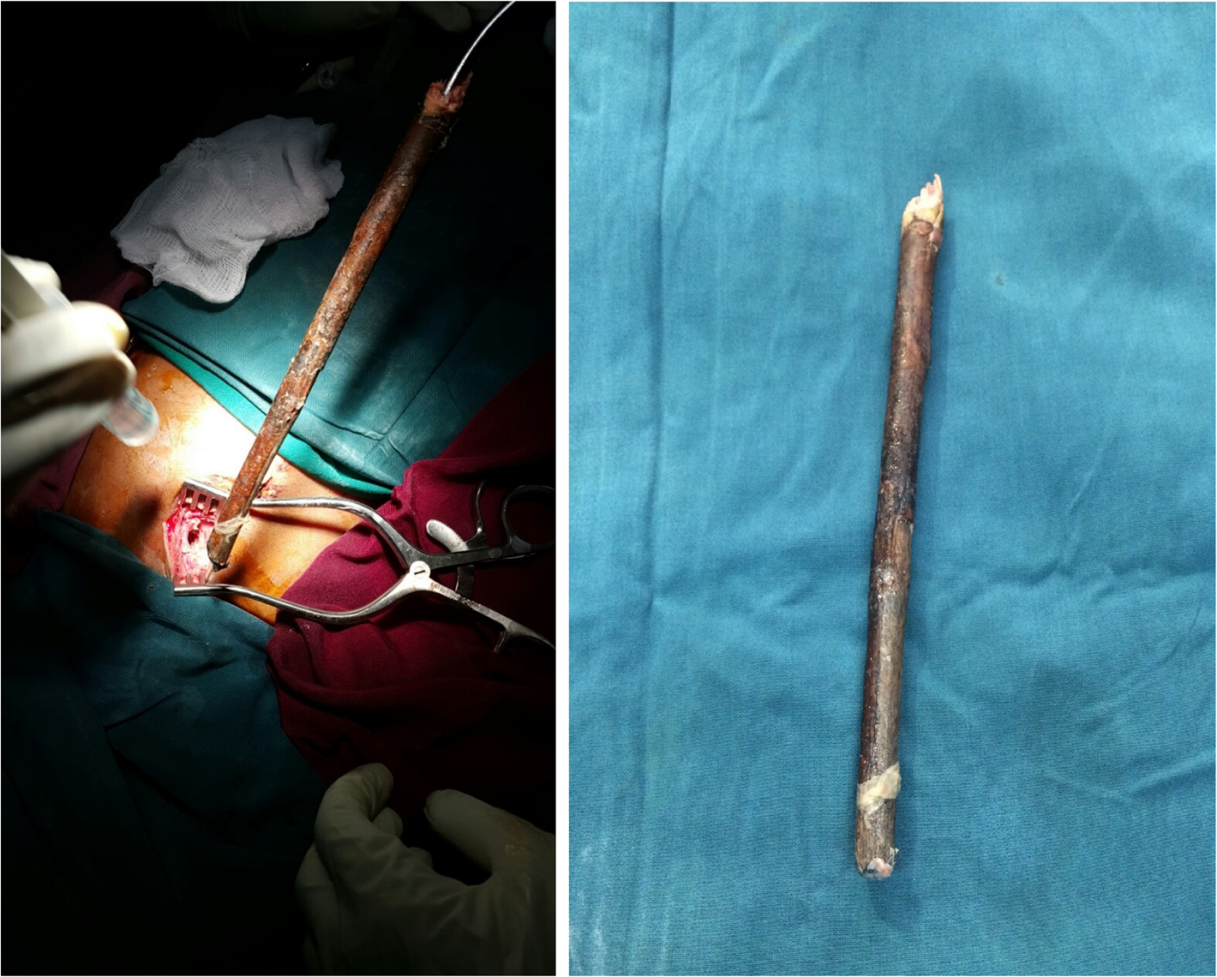
Inside the operating room the wound was widened and impaling object was gently pulled out

He was then taken to the operating room and put under general anesthesia. The skin surrounding the stick was incised and surrounding tissue was dissected. The stick was removed with gentle traction, in the line of the trajectory, without performing a thoracotomy. (Fig. 2) Vital signs remained stable and there was no major bleeding. He had a smooth post-operative course in hospital. Control chest x-ray showed well expanded lungs. Chest tube also drained minimal serous fluid and was removed before discharge on the 5th day. He was seen at the clinic at 1 week, 1 month and 6 months following discharge but no complications were detected.

### Case 2

A 12 year old boy presented after 24 h of sustaining thoracic impalement injury. He fell off a two-wheeled carriage and landed on a stick with his chest. He was immidiately taken to a local health center where chest tube was inserted and he was refered to our hospital.

**Fig. 3:**
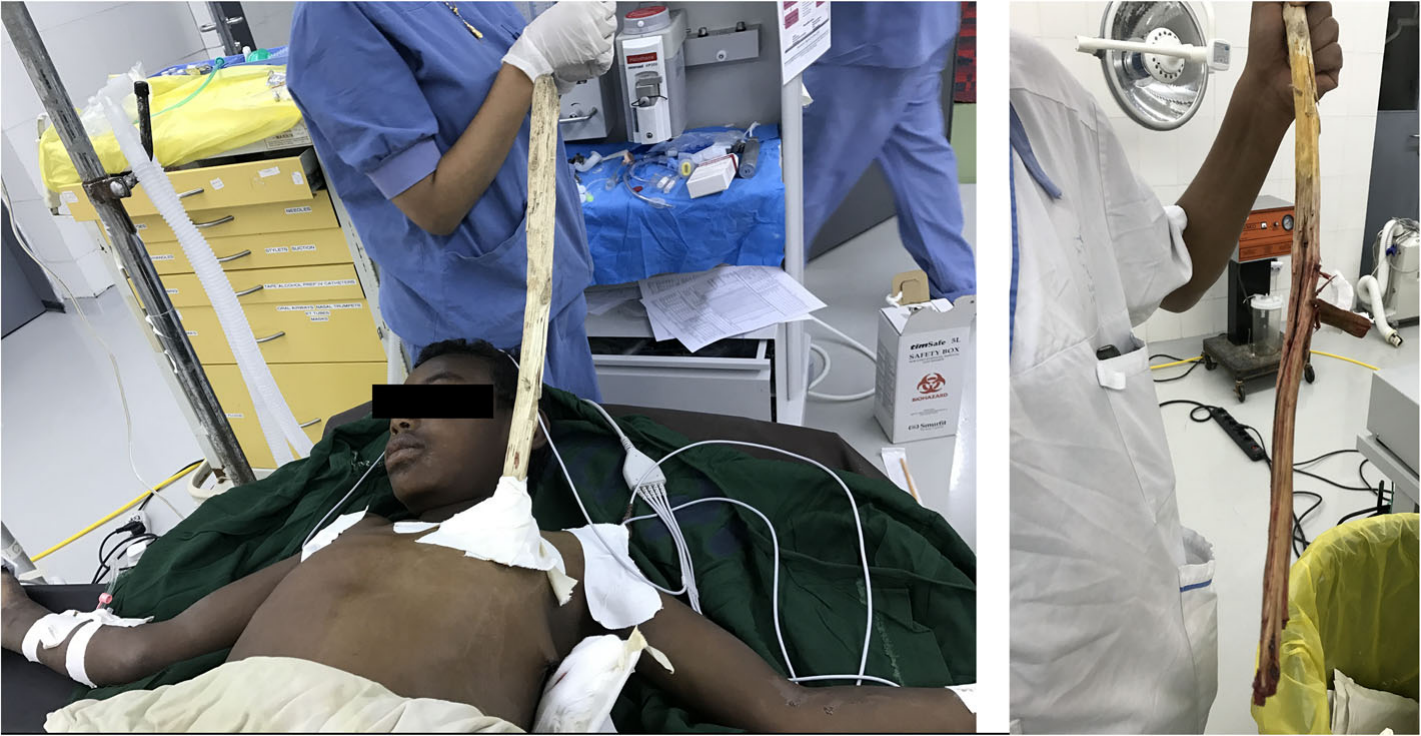
The chid presented with a huge length of the impaling object on the left anterior chest. The stick was stabilized by a bulky dressing around the entry site. Image taken after removal also shows the significant remaining length that was inside the body

**Fig. 4:**
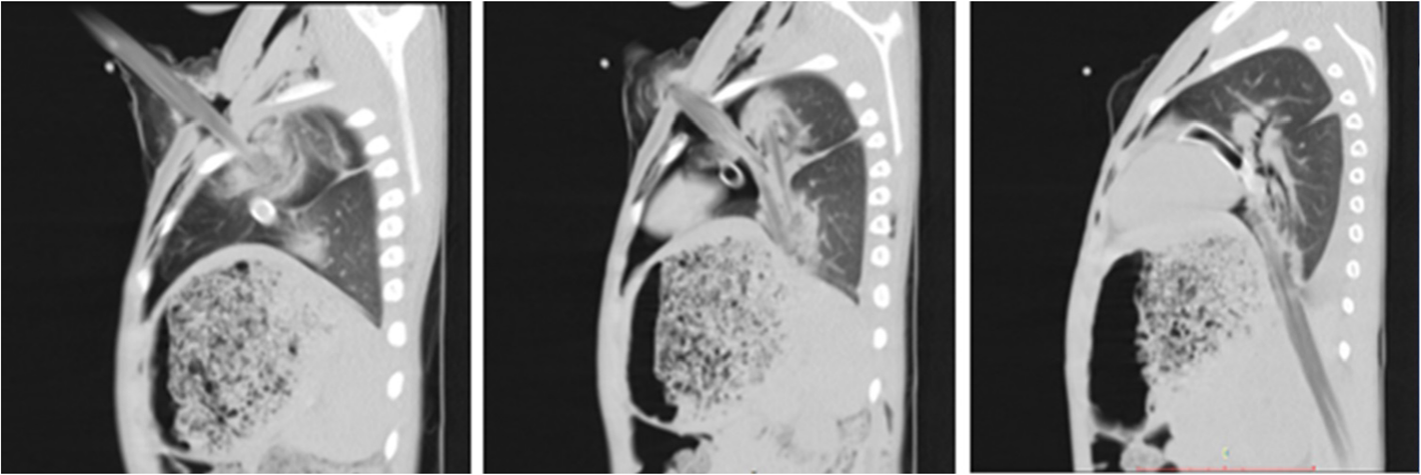
Chest CT showing trajectory of the impalement in the sagittal plane, passing in close proximity to hilum of the lung and the heart. Most inferior extent seems to penetrate the diaphragm

Upon arrival, there was a huge stick impaled in the left hemi-thorax with a diameter of 3.5 cm and around 50 cm length visible outside. It had entered the chest directed obliquely downwards at the mid-clavicluar line of the 2nd intercostal space and there was no exit. (Fig. 3) He had mild tachypnea, chest tube drained 150 ml blood and air entry over the left hemithorax was decreased. His other vital signs were normal and there were no injuries detected in other systems, including the abdomen. CT scan revealed a long trajectory extending through the lung just posterior to the hilum towards the diaphragm. (Fig. 4).

He was taken to the OR and explored through a left anterolateral thoracotomy. The stick was going through the right lung and partially penetrating the diaphragm. The major vessels and airways were intact. An upper midline laparaotomy was also made to inspect the diaphragm and reveled no penetration from the abdominal side. The stick was slowly pulled out and lung injury was repaired. There was no major bleeding. Postoperatively he had a smooth recovery and was discharge on the 7th postoperative day.

## Discussion and conclusion

Thoracic impalement is rare [4]. There are only a handful of case reports describing it in children. As the number of patients is low; many rely on experience from treating adults [2, 3]. Here we try to review the limited literature on isolated thoracic impalement in children and discuss our experience. (Table 1) Reports of thoracoabdominal impalement are excluded.

**Table 1:** Reports of thoracic impalement injuries in children

Author/year	country	Age	Sex	Impaling object	Mechanism of injury	Injured organs	Surgical Approach	outcome
Asch, 1974 [15]	USA	8 year	M	Radio aerial	Fell off garage roof	Passed mediastinum, Subclavian vessels compressed	Sternotomy, Thoracotomy, supraclavicular	uneventful
Massad 2002 [16]	USA	16 year	M	icepick	Street fight	Right Ventricle	Thoracotomy	uneventful
Edwin 2009 [7]	Ghana	18 year	M	Gun barrel	Faulty rifle recoil	Right lung	Thoracotomy	uneventful
Edwin 2009 [7]	Ghana	17 year	F	Umbrella spoke	Assault	Aortic injury- pseudoaneurysm	Thoracotomy	Uneventful
Riggle 2010 [3]	USA	15 months	F	knitting needle	fell off her car seat	Right lung	Thoracotomy	uneventful
Gettig 2015 [2]	USA	4 year	F	knitting needle	fell off couch	passed mediastinum without injury	Sternotomy	uneventful
Kulaylat 2015 [17]	USA	2 year	F	nail	Self-inflicted	Right ventricle	Sternotomy	uneventful
Our report	Ethiopia	7 year	M	Wooden branch	fell off a tree	Passed mediastinum- Right and left lung	Local exploration	uneventful
Our report	Ethiopia	12 years	M	Wooden branch	fell off a Carriage	Left lung, diaphragm	thoracotomy	uneventful

### Mechanism of injury

Impalement in children are usually due to falling down accidents in the home setting [14]. From our case review, the younger children demonstrate this pattern while the older adolescents had fight injuries. (Table 1) The exception was one toddler who sustained a self-inflicted injury while at a construction site [17].

Most of the cases reported are from the USA and only two case reports from Africa. (Table 1) The cases from this report are different in that they occurred in older children from a rural setting, impaled with large wooden sticks while playing outside. Similar mechanisms of injuries have been reported in abdominal impalement injuries from villages in India [18].

### Care at the scene and transport

Any attempt at removal should be avoided in all circumstances. One of the major challenges is releasing the fixed impaling object, especially when it is huge. Sometimes this entails cutting the object for deliverance or reducing it to a manageable size for transportation [4, 11, 13]. The traumatic agent can also be mobile against the injured person. It has to be stabilized to avoid aggravating the injury. The commonly used method in the field is applying bulky dressings around the exposed part of the object [3, 5] Patients have to be transported quickly but a large impalement may create difficulties [8]. Sometimes the usual supine position will be impossible and lateral positions would be appropriate [5].

All these safety measures relay on availability of paramedics who are called to the scene, ready and competent with all necessary equipment. From the cases we described, it can be inferred how poor the health care system is. There is no medical personnel or ambulance to come to the scene of the trauma. Meanwhile the villagers even had repeated attempts at removing the objects by themselves before they finally brought the child to a health center. Positioning during transport was not as such difficult because the impalement wasn’t through and through in both cases. However, referral to our center was delayed 24 h because of the distance needed to travel.

### Trauma care

On initial presentation, the dramatic nature can be distracting [14]. However, it should not be forgotten that these injuries have a component of blunt trauma due to the high force required to produce such an injury [3, 4, 8]. We should conduct a thorough examination to exclude subtle associated injuries. This includes monitoring for airway compromise or cardiovascular instability [3, 8].

Since the initial trauma care was delivered at another center, we cannot asses its adequacy. However, we can at least see that life saving measures such as chest tube insertion was provided prior to referral. Tetanus and antibiotic prophylaxis were also administered as the impaling objects have high infective potential [4, 13].

### Imaging and pattern of injury

Patients usually require immediate surgery to remove the traumatic object. However, if the patient is hemodynamically stable, imaging can be obtained to comprehend the full extent of injury and plan the surgery [4, 18, 19]. CT scan, particularly CT angiogram is the preferred modality [2]. The challenging aspect may be a huge impaled object that makes radiologic examination impossible [5].

On the other hand, sometimes the impaling object may be concealed if it lodges inside the body in its entirety [18]. We found two such cases in our review. In both cases there was fight/assault but patient was not aware of impalement. Subsequently this led to a delay in presentation and a diagnostic dilemma [7, 16].

As it is a penetrating trauma, the degree of damage depends on the trajectory of the impaling object [8]. When the chest wall is penetrated, the lung is the most frequently injured structure followed by the heart (right ventricle) [1]. As compared to adults, the incidence of thoracic vascular injury is very low in children. This is in alignment with accepted differences in pediatric physiology including increased vessel elasticity [2]. It can also be seen from our first case that even while traversing the mediastinum, no vessels were injured.

### Approach to removal of impaling object

It is recommended to attempt removal in the operating room where there is instrumentation and personnel able to deal with all types of intrathoracic injuries [4, 8]. Cardiopulmonary bypass should be on standby in case of major vessel or cardiac injury [4, 7]. Positioning should also be taken into consideration if impalement poses difficulty during induction of anesthesia [11].

The main fear with removal is loss of tamponade effect and subsequent hemorrhage that may follow [4]. Different approaches have been used depending on preoperative imaging. A foreign body superficially penetrating the lung without mediastinal traverse in an otherwise stable patient, can be removed in a controlled setting [3, 12, 19]. If the trajectory is in the subcutaneous tissue, fistulotomy like incisions may be made along the entry and exit site to aid removal [11, 20]. Other thoracic impalement should be removed under direct vision which entails a thoracotomy or sternotomy [2, 7, 12]. Recent reports in adults have also demonstrated successful use of video assisted thoracoscopy [21].

Most of the cases described in our review had either deep penetration of the lung, mediastinal traverse, vascular or cardiac injury necessitating thoracotomy/sternotomy for removal. Only one impalement from this report was removed without direct vision. We decided to deviate from this principle even though the child had mediastinal traverse, because of the trajectory on CT scan. It entered the superior mediastinum on the right side, behind the sternal notch, and exited mediastinum superiorly fracturing the left clavicle. With the assumption it was away from the heart and major vessels, it was slowly withdrawn and there was no bleeding. All other reports in our review also had a good outcome. This might be because patients able to reach the hospital are relatively stable.

In conclusion impalement injuries are very rare, complex and challenging scenarios. Additional challenges we observed managing these children in a developing country include lack of paramedics, lack of tertiary care centers or fast transportation, unavailability of sophisticated imaging and cardiopulmonary bypass. Our children were lucky as they escaped this potentially fatal injury, even in the absence of some of these precautions and facilities.

## Data Availability

Additional information regarding the study can be obtained upon request of the corresponding author. Contact via email- negashsamie@gmail.com
